# What Are the Most Reliable Symptoms and Signs of Approaching Insanity

**Published:** 1896-02

**Authors:** D. R. Wallace

**Affiliations:** Waco


### TEXAS MEDICAL JOURNAL.

ESTABLISHED JULY, 1885.

PUBLISHED MONTHLY.—SUBSCRIPTION $2.00 A YEAR.

VOL. XI. AUSTIN, FEBRUARY, 1896. NO. 8.

### Original Contributions.

For the Texas Medical News..

### WHAT ARE THE MOST RELIABLE SYMPTOMS AND SIGNS OF APPROACHING INSANITY.

BY D. R. WALLACE, M. D., WACO.

[Read before the Central Texas Medical Society, Jan. 15, 1896.]

THAN this, the subject designated as the one on which it is desired I should prepare a paper to be read at this meeting of your body, I know none connected with the general subject of mental unsoundness of more importance; none, an acquaintance with which is likely to stand the general practitioner in better stead in discharging his duties to his clientelle.

How many a poor victim of this terrible malady has sunk down into hopeless mindlessness, into intellectual darkness, uncheered by one mental ray of light, simply because the family medical adviser, in ignorance of what he should have known,—what he had the means within his reach of knowing,—did not interpose his offices until too late. In view of the importance of a knowledge of mental troubles in his daily work, I have been amazed at the ignorance of the average practitioner. G. Fielding Blandford, Fellow of the Royal Society of London, a lecturer on psychological medicine at St. George's Hospital, does not use words too large, nor language too strong, when addressing his students he says: "It is a branch of our art ever forcing itself upon our attention. There are diseases described in the lectures you here attend which throughout your whole lives may never come before you. You may never see a patient with hydrophobia; you may be so blessed with a healthy locality you may never have to treat Asiatic cholera, or ague and other ailments due to locality or special occupation you may never witness; but fortunate indeed you will be if you spend your years in practice without being called on to treat, or to express an opinion upon some case of unsoundness of mind. Your female friends, after parturition, will be attacked with insanity; their boys and girls, as they come to puberty, will show symptoms of it; at the climacteric of life men and women will break down, and in old age insanity will merge into fatuity and dotage in the general decay of mind and body. From the cradle to the grave, the mental, no less than the bodily health of your patients will be your care; and not only will you have to treat them, you will have to send them away from home under legal restraint; to plead their irresponsibility in courts of law, if in their frenzy or folly they commit crime; and when they are dead you will be called upon to testify to their competency or incompetency to make the will they have left behind."

With the elder Flint, it is a growing conviction with me that as the profession shakes itself free from superstition and unreasoning nonsense, engendered by ages of ignorance, as "the minds of doctors are widened by the process of the suns," the medicine of the future will become in its aims and objects more and more preventive. "Preventive medicine," says Daniel Hack Tuke, "is justly regarded as the highest department of the medicine of the future, prophylaxis being a higher aim than therapeutics." I know of no disease to which humanity is liable in which the timely aid of the physician can be of more avail; none in which the field of observation is so broad, and the circumstances so numerous and varied as in mental maladies. All literature is full of instances of distinguished men threatened with this dire calamity; upon the ragged edge looking forward to its dreaded possibility. To mention names were needless. All with a tolerable acquaintance with English literature even, will not fail to recall scores whose lives were rendered a ghastly night-mare by this horrid apprehension. Euripides, the most tragic of the Greek tragic poets, has two lines, which, literally translated, mean "When the Divinity means evil for a man he first strikes his mind," which passed into the Latin proverb: "Whom Jupiter would destroy he first makes mad." Says an authority, not indeed as Homer said, "If I had ten tongues and ten mouths could I describe adequately the mental sufferings, the dire despair, the agony of fear, the terror of impending, though imaginary troubles, which many of the insane endure."

There are the fewest diseases that to the eye of the educated physician give such timely warning, and that, too, by unmistakable indications, as mental maladies. It is true, to be sure, some come unheralded, but these are exceptionable,—so exceptionable you might pass your whole professional life without seeing one of them.

But before entering upon an enumeration of the signs and symptoms that herald an approach of insanity, it were well, perhaps, to note some of the more efficient predisposing causes, as leading up to, by easy approaches, and explaining the symptoms of the coming storm.

I may remark in passing that a theory was put forth some years ago by a celebrated German alienist, that all mental unsoundness is the result of sin and wickedness,—a theory now departed to the limbo of forgotten things. The best fall victims, the worst escape it.

Whatever theory of mind may be held, whether it be regarded as a distinct entity or the result of material organization, the brain is the organ without which there can be manifestation of its activity, or even of its existence. Therefore it is subject to the laws of physical life in general, and of those of cerebral life in particular. I need not consume time in pointing out what must have been noticed by all who have been thrown much with the insane,—what numbers of them have odd, ungainly movements, misshapen bodies, ears, sexual organs, teeth,—but I will call attention especially to the shape of the skull, which is sometimes below, sometimes above the average size. The largest skull and heaviest brain on record were those of a negro man who died some years ago in one of the Virginia asylums. There was nothing peculiar about his history, except that he was a criminal from his cradle. It is the shape of the skull to which I call attention,—the frequency of which we find brachycephaly and dolichocephaly, a flattening of one or the other of the cranial ovoid; the disproportion between the base and the top of the skull, with various other deviations from what is regarded as a typically shaped skull. Foville found one-sixth of the insane who came under his observation with misshapen skulls. Although congenital, these abnormally shaped skulls are not necessarily inherited though they generally are. But the most common and characteristic peculiarity among the insane is a symmetry of the cerebral convolutions or hemispheres, in gyral bulk, development and arrangement, defective in interhemispheric communications, —technically called porencephaly; abnormal development of ganglia, conducting tracts in vascular system.

Much more might be added to these physical signs indicative of tendency to mental deterioration. When observed, the medical man is forewarned. It were easy for one who has lived under the same roof with the insane as long as the writer to fill a paper much longer than it would be proper to read in such presence, with instances of these abnormalities, but I tax your patience with but one. It may inteerst if it does not instruct you. There was admitted into the North Texas Lunatic Hospital at Terrell, in 1886, an individual, said to be a woman, dressed as a woman,—a wife. The husband accompanied her to the institution. He was an imbecile but one remove from an idiot. Regarded and dressed as a woman this patient was placed on the female side of the house. A thin beard was scattered over the face. The average make-up, hips, chest, arms, were those of a man. There were no mammary glands. For the female patients with whom she was thrown she showed no interest, but upon more than one occasion showed some erotic emotions towards one of the female attendants. Dying of tuberculosis, the autopsy showed labia, greater and less, lined with mucous membrane, but not communicating with any vagina,—a clitoris in shape of a rudimentary penis,—the meatus of the urethra just under, but no vagina, no uterus, no ovaries. There were rudimentary testes, just where lodged I don't recall. Was this a man or a woman? Certainly much more the former than the latter. The husband was devoted. Walked from Navarro county several times to visit the so-called wife. Some months before her death he got work in Terrell at a restaurant. When the man-wife died he refused to eat and died of grief.

To the practiced, educated eye of the experienced alienist, there are certain psychological indications of a depraved degenerate neurotic condition, threatening mental integrity, of easier interpretation, perhaps, than the physical signs enumerated above. The most noteworthy of these are a self-centered, self-regarding disposition, so pronounced as to lead the subjects of it to be wholly oblivious of the comfort, convenience and happiness of others,—intellectual twists seeming to disqualify for looking at life, its duties and pleasures, as other people, moral obliquities, rendering them insensible to all moral distinctions,—they have no idea of right and wrong. As children, they show little or no interest in the sports and amusements of other children. They seem to dwell apart. They amuse themeselves in odd ways, not seldom in the infliction of pain on the lower animals, or on children smaller than themselves. Though cunning and shrewd, they are most generally incapable of continuous thouh They are averse to trying to learn,—will not attend school if they can avoid it; frequently play truant; wander about the woods and fields, diverting themselves in the most irrational ways. Devoid of taste and pride, they are generally indifferent as to their personal appearance, and to the opinion of their fellows. They are generally prigs, helping themselves to whatever they desire when they can do so without being detected. Asked why they do naughty acts, they reply, as a general thing, they don't know, or because they want to. There are but two issues for these degenerates. They are sure to fetch up at one or the other, the penitentiary or the lunatic asylum. In order to emphasize this degenerate organization, I have called attention to the worst form of it. There are all shades and degrees, from the slightest departures from a normal condition to the most degenerate neurosis. The family physicians are not seldom consulted by the parents of these degenerate specimens of humanity, as to what is best to be done in the line of education and employment, in order to insure future well-being, and he should be prepared to advise as to the most effective methods of counteracting the tendencies of these vicious organizations. This is "the insane root that takes the reason prisoner," in that numerous and diversified class of insanities formerly known as the monomaniacs, but better known in the nomenclature of mental unsoundness as "paranoia." They are legion. They furnish the insane suicides and murderers of our times,—social world-menders who are quite sure all our institutions are founded in injustice,—that all things are out of joint, and that God has called them to the work of setting them right. Religious fanatics, who, though it often happens, are the most abandoned moral wrecks, yet feel they are commissioned of heaven to bring the world to the worship of the true God, in doing which there is no crime they may not commit. History is full of their crimes and misdeeds. The Joans of Arc, the Guiteaus, Francois Ravaillac, the murderer of

Henry the IV. of France, Guy Fawkes, dominated by a religious idea, they become assassins of persons in authority, believing doubtless they are doing God's service, executing His will. To these are to be added pyromaniacs, kleptomaniacs, sexual perverts, mysophobiacs, claustrophobiacs, agoraphobiacs and hundreds of others affected with nameless forms of mental troubles, having their origin in a faulty organization. All may be classed together as paranoiacs.

It is in regard to this class the advice of the judicious physician is of inestimable value. Many persons born with degenerate tendencies, have been saved to themselves, their families, and society, by the timely advice of the skillful physician. Did it not smack too much of self-praise to make it in taste to do so, I could give, not a great many, but some instances, in which a course of life, adopted at my humble suggestion, has resulted in the greatest good, saving persons who are now useful members of society from a fate infinitely more to be dreaded than death.

The most eminent authority in this country or Europe (at least such I regard him), Prof. Henry Maudsley, holds this language: "From time to time you may see two persons who have the same faulty heritage, and who, so far as we can judge, have not differed much in the degree of their predisposition to insanity, go very different ways in life,—one perhaps, to reputation and success, and the other to suicide or madness. A great purpose earnestly pursued through life, a purpose to the achievement of which the energies of the individual have been definitely bent, and which has, therefore, involved much renunciation and discipline of self, has, perhaps, been a saving labor to the one, while the absence of such life-aim, whether great in itself, or great to the individual in the self-discipline which its pursuit entails, may have left the other without a sufficiently powerful motive to self-government, and so have opened the door to the perturbed streams of thought and feeling that make for madness." He adds, in regard to self-control, these remarkable words, "There can be no doubt in the capability of self-formation that each one has, in a greater or less degree, there lies a power over himself to prevent insanity. Not many persons need go mad, perhaps, at least from moral causes (what the author here means by 'moral causes' included the whole ætiology of disease, except purely physical ones), if they only knew the resources of their nature, and knew how to develop them systematically."

We have now reached a vantage ground, in which we are in a position to watch the signs and symptoms of approaching insanity with a proper understanding of their nature and meaning. Does this fell malady generally give any intelligible indications heralding its approach? I am in a position to answer "Yes." The paranoias all do. Acute mania and acute melancholia are generally preceded by symptoms, which, though less intelligible and decisive, are enough so to put the experienced medical man upon his guard. Dementia is usually the terminal phase of many forms of insanity; but there is what is known in regularly systematized treatises on this subject as "acute dementia," which, with mania-transitoria, gives, so far as I know, no warning of its approach. Puerperal mania, in my experience, gives little or none. The relatives of the victims of acute mania and melancholia, when conversed with in regard to the history of any particular case, will say, with the fewest exceptions, that they noticed that he or she was not just like he or she had been aforetime; that he or she was queer; was this, and that and the other; that they now know it was insanity in the coming on, but they suspected nothing of the sort at the time. This was the time for the family physician to have rendered assistance. In most cases of this sort timely treatment would have been effectual, whereas, when the disease fully develops, it is always doubtful whether a cure can be effected.

Paranoia, a Greek word transposed to English, unmodified even by an English termination, was used by Plato, Aristotle, and the Greek tragic poets, Euripides and Æschulus, to mean madness in general, just as we use the word insanity. It is made to do duty now as a name of various forms of insanity. Perhaps no two authors use it just alike, or apply it to the same forms, phases and stages of mental unsoundness. Landon Carter Gray, whose work is just from the press, makes a different use of it from any previous writer. Hammond, who wrote on insanity twelve years ago, does not use it at all, using monomaniac, or reasoning mania in place of it. Dr. Gray's definition is the best I have seen, and differs but little from Spitzka, who wrote his eight or ten years ago. But the former classes certain forms of insanity as paranoia; the later classifies them differently. With Dr. Gray, all mental disease, characterized by logical or systematized delusions of persecution and self-exaltation, without excitement or impairment of memory, is paranoia. This is making it too inclusive, I think, but I have no time for discussion. But to our subject proper, "What are the most reliable signs and symptoms of approaching insanity?" This is the question of questions. The question interesting the general practitioner above all others. In cases of traumatism, the symptoms are obvious; in cases of certain mental unsoundness known as "crankiness" it is difficult to draw the line as to where the crank ends and the insanity begins. Guiteau was a typical case of this sort, and had he lived a few years more, none could have questioned his insanity. To dispose of this form of madness in as few words as I may, let me say, when the exaltation and depression become more and more marked, and the suspicions more and more baselesss, so as to make it impossible for the patient to keep himself in harmony with his surroundings, know then that the unfortunate man is doomed if he does not get relief, and that speedily, although, perhaps, nine out of ten of his neighbors would swear that he is not only not insane, but shrewder than they ever knew him to be. And it is a fact, in certain persons; when their minds become concentrated upon their persecutions and delusions, they seem shrewder than they formerly were.

In lipemania, or melancholia, it may be noticed long before the patient breaks down that whereas formerly he was hopeful, not easily discouraged, lately he looks on the dark side, is easily discouraged, much to the astonishment of his friends. When this has gone on some weeks, or months, perhaps, he begins to have fixed delusions and suspicions,—perhaps has been guilty of the unpardonable sin, is lost; but unlike the paranoiac, who thinks everybody but himself at fault, the lipemaniac thinks nobody at fault but himself. When he reaches this stage, he should be placed under restraint at once, as there is always danger of suicide. The paranoiac may commit suicide, but is much apter to commit murder. The melancholiac may commit murder, but is much more liable to commit suicide.

It is a little singular, but a fact known to all alienists, that acute mania commences in the same way as lipemania,—that is, the symptoms of unnatural depression, which may last one, two, or even three or four weeks, but here the two part company,—the lipemania goes on down, the depression deepening and intensifying; the maniac, to the delight of his friends, reacts, regains his spirits, is all right for some days,—but soon it is observed he has too much spirit. When told he is not drinking at all, they are puzzled, but the subject goes on, becomes more and more excited, and soon the unwelcome truth bursts upon his friends, that he is an acute maniac.

There is a form of insanity named by the French "mania circulaire," folie a double form, by the English as recurrent mania, which may be heralded by the same symptoms as acute mania and lipemania, being both, first one and then the other, with lucid intervals. It is needless to say more of this form of mental unsoundness than simply to add: I am not aware of a case of it ever recovering; certainly none under my observation ever did. May get apparently well a dozen times.

There is another form of mental unsoundness equally hopeless. Though it has many names, it is perhaps better known to my hearers as paresis, or general paralysis of the insane. From the moment a single symptom of this fell malady makes its appearance, guided in my prognosis by my reading and experience, I would say the case is hopeless. Like Crichton Browne, I thought I had cured a typical case when superintendent of the asylum at Austin. I discharged the case cured, but he died of the disease all the same, two years later.

The disease is ushered in with very unmistakable symptoms. It generally attacks, unlike most mental troubles, the well-to-do, free-livers. I have never known a case among the hard working poor. Cases of the sort, I believe, do occur, but I have never seen one. Though hopeless *ab initio*, it may be well for the general practitioner to recognize its presence at the earliest moment. The diagnosis is not difficult. There is no mental malady whose presence it is so necessary to recognize at the earliest moment possible as paresis. The victim of it, though possessed of hundreds of thousands of dollars, may make himself a financial wreck before his family or friends even suspect anything is the matter; hence the necessity of the medical adviser to be on the alert. If he is so, in most cases, not in all, his intervention at first may be of vast importance to the family; in fact may save them from beggary.

When one who has been a cautious trader engages in reckless speculation; when from being truthful and sober he becomes, all at once, an unblushing liar and a heavy drinker; when from being modest and temperate in his talk he becomes obscene and profane; from being chaste and measured in his conversation he becomes a man about town, spending much of his time and squandering his money with disreputable persons, boasts of his thousands when it may be he does not own hundreds, when he has hundreds of ships upon the oceans, visiting every port, and herds upon a thousand hills; when he is a great warrior, knowing more of the strategies of war than Alexander, Hannibal, Cæsar, or Napoleon, has more physical prowess than Hercules or Achilles, not to mention such pigmies as John Sullivan or Jim Corbett; when from being a quiet, well-dressed gentleman, observing all the proprieties of life, he becomes boistrous in his talk, careless in his dress, and insensible to public opinion in regard to decorum, know, my brother, what you have to deal with, and act at once upon your conviction, before this unfortunate has ruined himself and family, if he has not already done so. You may save his family; for him you can do nothing except to have him restrained in an asylum. Trades he has already made, or obligations incurred, you may have declared null and void, and thus save to his estate thousands of dollars. You may pass through your professional career, and never see a case, I have seen but two cases, among over three thousand patients under my care. I have seen great numbers in Northern asylums, when, at the time, with as many patients under my charge as in the North, in which there were dozens, there would not be a single one. Why there should be such a disparity between the South and the North in this regard, I have no explanation. It does not seem to be in the least hereditary. A word as to one of the cases above alluded to as having been under my charge. I will not mention his name, but only say his daughter is well known and dear to all Texans. He was a scholarly man, and observed to me one day, when unable to walk without assistance, that Hercules and Sampson were greatly overestimated for their strength; that he had more strength in his right arm than they had in their whole bodies, but held out his left arm. On another occasion, he remarked that the money of the Goulds and Vanderbilts was a mere baggatelle compared with his; that he was really the richest man in the world; that he was, at that time, constructing a break-water from Florida reefs to Vera Cruz, which the engineer had estimated to cost five billions; "but," he added, "I have concluded, upon reflection, as the work has progressed but little this side of New Orleans, to stop it at Galveston, and pay the national debt of the United States with the other two billions." He had already paid, in fact, our national debt, except forty millions, which he said he had left unpaid just to show the country how difficult it would have been to pay the whole. Turning to a Norwegian by the name of Johnson, his shadow, another patient as crazy as himself, whose assiduous attention to the "old gentleman," you may call him, was something to behold, "Reed, you know all about it; tell the doctor if what I say about how much I am worth is not true." "Reed," or Johnson, rather, replied: "Yah, doctor, it is every verd true. I see de doctor pay de monish." This man Johnson lived in Waco before he became insane. His wife and children may live here still. He supposed he was receiving one hundred dollars a month, which the doctor was sending to his family, for his services. A more devoted creature I never saw. The old man could not move, day nor night, but what he was at his elbow, ready to render him assistance. This case may serve to impress upon the hearer the nature of general paralysis of the insane.

I close with a remark or two for a purpose. We hear much of what is set down in the reports of lunatic hospitals as "religious insanity," the average being from ten to thirteen per cent. Now I want to say here, and now, that I do not believe that "true religion, before God the Father," ever was or ever will be the cause of insanity to any human mind. Religiosity and Theology doubtless have counted their victims by the thousands. Religion impels to do unto others as we would have them do unto us; to visit the fatherless and the widows in their affliction; keep ourselves unspotted from the world. In other words, it means an understanding of our true relations to the world in which we are placed. In still other words, it is a feeling of Altruism, or care for our fellows, which never did, and never will, while the world stands, produce a single case of insanity. That unfortunate paper, read by me before the T. S. M. A., at its last session, at Dallas, and at which certain bigots and zealots affected to take offense, meant just this, and nothing more.

A most respectable religious journal, published at Nashville, Tennessee, says of that paper in its May 16th issue: "Dr. Wallace may be much nearer the truth than religious institutionists are willing to admit, when he holds that institutionism in religion is a fruitful cause of insanity and crime among the people in Christian nations. He might have gone further, and suggested that it is a prolific cause of skepticism, agnosticism, infidelity and atheism in this country." The editor adds: "The substitution of religious institutions for New Testament Christianity has unbalanced the people in both mind and morals, by causing them to rely for their development upon defective and inefficient institutions, instead of depending upon the 'simple teachings of the Christ' for spiritual nourishment. The 'Theoretical Reflections' of Dr. Wallace on this point, strikingly resembles the reasoning of Paul along the same line. Paul puts it thus: 'When they knew God, they glorified him not as God, neither were thankful; but became vain in their imaginations, and their foolish heart was darkened. Professing themselves to be wise, they became foolish.' "

One of, if not the greatest writer on insanity, living or dead, whose authority I have already invoked,—I need hardly say I refer to Prof. Henry Maudsley,—says, in his work upon "Responsibility in Mental Diseases," page 294: "Is it any marvel, then, that the theoretical recognition of a higher aim in life, which they (hypocrites) make once a week as a conventional duty, has no real informing influence in the formation of character; that it is a doctrine which, by an easy self-deception, is held on the condition of its being a sort of sleeping partner, and taking no part in the management of affairs? No argument is needed to prove that it must be hurtful to the intellectual and moral nature to hold a belief on such terms. * * * It is the same with another great interest of life, which, were it as real as it is reported to be, should exert a most powerful influence upon the development of the mental nature, viz.: religion. A majority of men discharge its duties automatically, accept its doctrines formally, paying to these a lip homage, without having a distinct grasp of them, or even pursuing them in thought to their logical consequence. They believe vaguely, without ever caring to realize distinctly what it is they think they believe; are contented with a kind of belief which they would certainly at once repudiate in their worldly affairs. It needs no argument to prove that such a slovenly habit of thought is not only not conducive to, but it is greatly hurtful to mental culture; and that any mind which is content to hold beliefs on these terms is ill fortified by the development of its powers to exercise sound reflection on other subjects, or to react vigorously to the end under the burdens laid upon it. * * * Now, as ever and forever, it is true that the wrath, the folly, the madness of men, are made to praise Him whom sun and moon, fire and heat, winter and summer, mountains and hills, seas and floods, the green things of the earth, and the holy and humble men of heart, bless, praise, and magnify forever, but whom systems of theology, and the prophets thereof, have so much misrepresented and degraded. * * * For this we must not forget that, however clearly we trace the order of events, the mystery of their 'why' remains where it was; however clearly we may

"Follow one first matter through
Various forms and various degrees of substance,
'And in things that live, of life,
Until body up to spirit work,
In bounds proportioned to each kind,'

the power which determines why one tissue should supervene on another; why life should tend upward, which inspires and guides the everlasting becoming of things, must ever remain past finding out. Man himself, with all his sorrows and sufferings, with all his hopes and aspirations, and his labor wherewith he has labored under the sun, is but a little incident in the inconceivably vast operations of that primal, central power, which sent the planets on their courses, and holds the lasting orbs of heaven in their just poise and movement.''

